# Overview of research and practice progress in body composition measurement techniques

**DOI:** 10.3389/fmedt.2026.1789046

**Published:** 2026-08-28

**Authors:** Wenjuan Jing, Wenrong Jing, Shili Luo, Yinhong Chen, Qinghuan Guo, Juan Yang

**Affiliations:** 1 Department of Obstetrics and Gynecology, West China Second University Hospital, Sichuan University, Chengdu, China; 2 Key Laboratory of Birth Defects and Related Diseases of Women and Children, Sichuan University, Ministry of Education, Chengdu, China

**Keywords:** artificial intelligence, bioelectrical impedance analysis, body composition, computed tomography (CT), computer vision, dual-energy x-ray absorptiometry, magnetic resonance imaging (MRI), smartphone software

## Abstract

The measurement of body composition is one of the most important tools for evaluating an individual's health and nutritional status. Its field has developed rapidly in recent years due to technological advances and growing application needs. Currently, widely used measurement methods include dual-energy x-ray absorptiometry (DXA), bioelectrical impedance analysis (BIA), Magnetic Resonance Imaging (MRI), Computed Tomography (CT), and smartphone software based on Computer Vision (CV) and Artificial Intelligence (AI). Each method has its own advantages in terms of accuracy and convenience. However, these techniques still face problems related to measurement reliability and applicability across different populations. The purpose of this paper is to provide a comprehensive overview of the technologies, target populations, and research fields related to body composition measurement. Body composition measurement is an important part of health management, non-communicable disease management, clinical medicine, and sports and physical training. Along with new scientific advances, this article also presents potential future research directions in body composition measurement.

## 1 Introduction

Body composition analysis refers to the measurement of components such as fat, muscle, water, and bone in the human body. It helps assess an individual's health status, including disease states and other health conditions (1). Conventional measures like weight and body mass index (BMI) are widely used in clinical and research settings. However, they only reflect overall weight changes and do not provide accurate information on changes in body components. This limitation reduces the efficiency of current medical interventions (2). As chronic conditions such as obesity and metabolic syndrome become more prevalent, there is an increasing demand for more accurate technologies and assessment methods for body composition (3). This has promoted the use of various advanced measurement technologies. The purpose of this paper is to systematically review the technologies for body composition measurement, their target populations, and application areas, and to propose future research directions.

## 2 Major technical methods of body composition measurement

The main technical methods for body composition measurement each have unique characteristics, advantages, and limitations. The key information of the core methods is summarized in Table 1.

**Table 1:** Comparison of key advantages and limitations of body composition measurement methods.

Measurement method	Key advantages	Main limitations
Dual-energy x-ray Absorptiometry (DXA)	High accuracy and reproducibility	High equipment cost
		Potential radiation exposure
	Gold standard for bone mineral density and muscle mass measurement	
Bioelectrical Impedance Analysis (BIA)	Easy to use, non-invasive, low cost	Accuracy affected by hydration, electrode type, and op
	Suitable for bedside monitoring and broad screening	erator expertise
		Relies on population-specific predictive algorithms
MRI and CT	High-resolution imaging	High equipment cost
	Accurate for soft tissue measurement	Complex operation
		CT involves radiation
	MRI is radiation-free	MRI requires long scanning time and patient cooperation
CV and AI-based Smartphone Software	Convenient, low-cost, portable; Potential for remote use	Lack of long-term validation for tracking body composition changes over time
	High correlation with traditional methods (e.g., DXA) in body fat assessment	

### 2.1 Dual-energy x-ray absorptiometry (DXA)

Dual-energy x-ray Absorptiometry (DXA) uses the difference in absorption of low and high-energy x-rays as they pass through human tissues. This technique can visualize and quantify bones, fat, and lean body mass, and also assess bone density and fat deposition (4). In clinical and research settings, DXA is regarded as the gold standard for evaluating bone mineral density and muscle mass due to its high accuracy and reproducibility (5). It has a wide range of applications, especially in osteoporosis diagnosis and obesity-related research (6, 7). In addition, DXA's ability to assess body composition in children and adolescents is valuable, as it allows for proper monitoring of their growth and development (8). However, the high cost of equipment and the risk of radiation exposure limit its use in certain populations (9). In summary, DXA is currently the gold standard for body composition measurement and is irreplaceable in most clinical and research scenarios, but its use and limitations must be considered based on specific population and application circumstances.

### 2.2 Bioelectrical impedance analysis (BIA)

Bioelectrical Impedance Analysis (BIA) is a non-invasive technique used to measure body composition, including water and fat content. It works by evaluating the resistance of body tissues to a weak electric current (10). BIA is widely used as a clinical tool for large-scale screening and bedside monitoring because it is easy to use, non-invasive, and low-cost (11). This is particularly true for critically ill patients and those with cardiovascular diseases or cancer (12–14). However, the accuracy of BIA results can be affected by many factors, such as electrode type, operator skill, the subject's biological characteristics, and environmental conditions during measurement (15). In addition, changes in the body's hydration level can alter impedance readings, thereby affecting the estimated fat and lean body mass (16). BIA relies on specific predictive algorithms, so its accuracy depends on whether the used equations are suitable for the target population. There is a need for correction models applicable to children, young people, different ethnic groups, and populations with various diseases (17, 18). In short, BIA is a relatively simple and portable technology for body composition assessment, which is very useful in nutrition evaluation, disease monitoring, and public health screening. It should be noted that the accuracy and reliability of BIA can be affected by multiple factors, so it needs to be selected and used carefully with calibration for specific populations.

### 2.3 Magnetic resonance imaging (MRI) and computed tomography (CT)

The automatic analysis of body composition using imaging systems is gradually becoming a reality with the assistance of artificial intelligence and image segmentation algorithms. Magnetic Resonance Imaging (MRI) and Computed Tomography (CT) can provide high-resolution imaging, which is essential for measuring soft tissue composition. These techniques allow three-dimensional assessment of body composition and can distinguish between adipose tissue, skeletal muscle, and other soft tissues (19). MRI is an imaging technique based on the principle of nuclear magnetic resonance. It provides information on tissue structure and function without radiation exposure. MRI's ability to produce detailed images allows for continuous monitoring of fat distribution, muscle mass, and organ size (20). CT can quantitatively study body composition by measuring tissue density changes using x-ray computed tomography, which is very useful for assessing bone and fat tissue (21). Both MRI and CT imaging are highly predictive and useful in clinical practice. For example, they are used to predict treatment response and survival in cancer patients (22). Since MRI does not involve radiation, it is an ideal imaging choice for children, pregnant women, and those who need long-term follow-up (23). However, the high cost of equipment, complex operation, and radiation risk (for CT) limit their widespread use. MRI scans take a long time and require patient cooperation, and the characteristics of the magnetic field also impose restrictions on its use (24, 25). In addition, quantitative analysis using CT and MRI requires complex data and image segmentation, which needs professional personnel (26). In general, MRI and CT are accurate and reliable body composition measurement techniques suitable for research and high-end clinical use. However, their high cost and operational complexity limit their general application. It is expected that technological progress will reduce costs and promote their widespread use.

### 2.4 Ultrasonic techniques for body composition assessment

Ultrasonic techniques represent another portable and non-invasive approach for body composition assessment. These methods primarily rely on measuring the thickness of muscle and adipose tissue through ultrasonic echoes, which can reflect the corresponding components in the body (27, 28). This technique offers several advantages, including being non-invasive, radiation-free, low-cost, and highly portable, making it particularly suitable for bedside measurement and continuous monitoring in clinical settings (27, 28). For instance, ultrasound has been effectively used to predict body composition in premature infants within neonatal intensive care units, providing a convenient tool for monitoring their nutritional status (27). However, this method is highly operator-dependent, which can introduce variability in measurement results (28). Additionally, it is inherently difficult to measure deep body components with ultrasound, which limits its application in comprehensive, whole-body composition analysis (28).

### 2.5 Computer vision and artificial intelligence-based smartphone software

Recent technological advances, especially the improvement of Computer Vision (CV) and Artificial Intelligence (AI) technologies, have had a significant impact on body composition analysis software (29). Smartphone-based body composition measurement technology is becoming increasingly popular and is being continuously developed. This technique uses smartphone cameras and AI algorithms to measure various parameters, including body fat and muscle density (30). This new approach provides an intelligent and low-cost measurement option using optical imaging systems such as smartphone camera apps and 3D scanners (31, 32). Research shows that there is a strong correlation between smartphone-based two-dimensional image analysis and traditional methods such as DXA in body composition assessment, especially in body fat percentage (33). A study involving 929 adults found that the body fat percentage calculated by smartphones had a correlation coefficient of 0.90 with DXA, with lower error margins and better effectiveness than conventional BIA (33). In addition, the application of 3D optical imaging technology in smartphones has shown potential for use in military environments to monitor health and fitness, as it can independently measure anthropometric indicators such as waist circumference to estimate fat content (31). Compared with traditional body composition measurement techniques that require specialized equipment and face difficulties in large-scale application, CV and AI-enabled smartphone methods are convenient, low-cost, and potentially usable remotely, thus having broad application prospects in various fields (34). With the continuous improvement of AI technology, the method of estimating body composition using smartphones is expected to develop further and play a greater role in population screening and home monitoring. However, more validation is needed to confirm whether it can accurately track changes in body composition over time.

### 2.6 Other technical methods

In addition to the techniques mentioned above, there are various other methods for measuring body composition. The choice of method depends on the application scenario and required accuracy. Many experts consider the four-compartment (4C) model as the “gold standard” for body composition measurement (35). This model provides detailed information on the three main components of fat-free mass (FFM): water, protein, and minerals, based on standardized measurements of body volume, water content, and mineral content. However, the measurement methods required by this model are very complex and costly, making it difficult to apply in practice (3). Anthropometric techniques can be used to measure height, weight, skinfold thickness, etc. Although these techniques are relatively simple and easy to implement, practitioners need specialized training to obtain accurate measurements (36). Hydrostatic weighing, also known as underwater weighing, measures body composition based on Archimedes’ principle. However, its use is severely limited by equipment, infrastructure, operator proficiency, and time (37). In addition, it imposes great physical and mental pressure on participants. Recent technological advances have led to the development of systems such as air displacement plethysmography (ADP), which is more acceptable to subjects and has replaced underwater weighing (38). ADP assesses body density to determine body composition by measuring the volume of air displaced by the body in a closed chamber. It has been validated for infants aged 0–6 months and over 6 years old, but there are few studies on children aged 6 months to 5 years (39). It is a precise and easy-to-use measurement method, but the equipment is often expensive (40). In addition, the subject's hydration level and behaviors such as crying, talking, and exercising may negatively affect this method (41), making it less suitable for universal application. The isotope dilution technique uses isotopes to estimate total body water. Although this method is known for high accuracy, it is costly and complex to operate, so it is more suitable for research settings (42). Near-infrared interactance (NIR) uses near-infrared light to assess tissue composition in different media. Its advantages include speed, non-invasiveness, and portability. However, the accuracy of the instrument depends on several factors, including the model version, population-specific equations, measurement site selection, and skin characteristics (43). Therefore, it is recommended to use it cautiously in the general population (44).

## 3 Population applications of body composition measurement

Body composition measurement has important applications in different populations, and the key application points for major populations are summarized in Table 2. Beyond recognizing the importance of these measurements, understanding how to apply specific techniques in each population is crucial for deriving meaningful clinical and research insights.

**Table 2:** Key applications of body composition assessment across target populations.

Target population	Key application points
Athlete Population	Performance optimization for athletes
	Guiding personalized nutrition for athletes
	Athlete injury rehabilitation
	Assisting athlete selection and targeted training
Obese Population	Assess obesity phenotypes
	Predict metabolic risks
	Guide personalized weight management (focus on muscle-fat balance)
Infants and Young Children	Evaluate nutritional status
	Predict future obesity and metabolic disorder risks
	Guide healthy growth
Children and Adolescents	Assess nutritional status and disease risk factors
	Identify high-risk subgroups
	Guide intervention measures
Pregnant Women	Predict pregnancy complications (e.g., GDM);
	Guide nutritional interventions
	Explore maternal-infant health associations
Menopausal Women	Monitor fat increase and muscle loss
	Assess metabolic and cardiovascular disease risks
	Guide intervention to improve quality of life
Older Adults	Screen for sarcopenia
	Assess disease risk and functional status
	Guide nutrition and exercise interventions for healthy aging

### 3.1 Athlete population

Athletes are a unique group for body composition assessment because their body composition differs significantly from that of the general population. This difference arises from their specific training intensity, physical demands, and physiological adaptations (45). Therefore, the formulas used to estimate athletes’ body composition must also be specialized, which is particularly important when conducting body composition analysis for athletes. Studies have shown that predictive models for body fat estimation using ADP data based on athletes can improve accuracy and clinical relevance (46). For athletes, increasing muscle mass and optimizing body fat percentage are priorities, as these parameters affect their performance and competitive ability (47). During competitive seasons or intense training phases, athletes often experience a significant decrease in fat mass and a dramatic increase in lean body mass, but this pattern usually reverses during rest periods (48, 49). Regular monitoring of athletes’ body composition changes is crucial. Studies have found that the nutritional needs of young athletes differ from those of female athletes or master athletes. This difference emphasizes the importance of personalized dietary strategies to meet their specific needs for energy, protein, and hydration, as well as the appropriate use of sports nutrition supplements (50). To recover from sports injuries, it is essential to maintain or increase skeletal muscle mass while controlling the rapid increase in fat mass. Maintaining this balance is crucial for effectively restoring motor function and minimizing the risk of re-injury (51). In addition, the feasibility of genetic assessment has been re-examined. Genetic variations among athletes can significantly affect their body composition and performance, and some athletes have been found to have genotypes that are advantageous for athletic performance (52). Genetic data can provide insights for athlete selection and targeted training. Future studies should provide accurate and practical body composition measurement options suitable for the sport, gender, and age of athletes, and integrate genetic and nutritional strategies to improve athlete health and performance.

### 3.2 Obese population

Obesity is a global public health problem affecting people of all ages and economic groups, and it is closely related to a variety of chronic diseases (53). Assessing obesity solely by BMI is insufficient, because obesity is a complex and heterogeneous chronic condition. Traditional obesity assessment methods are increasingly considered inadequate for accurately reflecting an individual's health risks and metabolic status (54). With recent advances in body composition measurement technology, phenotypic analysis of obesity based on body composition has gradually become an important basis for personalized obesity management (55). BMI alone cannot distinguish between fat tissue and lean tissue, and it also ignores variations in fat distribution and muscle mass (including muscle composition and strength) (56). When body composition is analyzed in the context of different obesity phenotypes, it helps to diagnose metabolic risks more accurately than traditional BMI analysis, which overlooks important factors such as fat distribution and muscle mass (57). In clinical practice, techniques like DXA or CT are instrumental in phenotyping obesity, as they can precisely quantify visceral adipose tissue, a key driver of metabolic risk (58, 59). For broader screening and management, BIA devices that estimate visceral fat level and segmental muscle mass offer a more accessible, albeit less precise, alternative for tracking changes over time (55). For example, studies have shown that postmenopausal women in the simple obesity group have higher bone mineral density, while those in the sarcopenic obesity group have much lower bone mineral density and a higher risk of osteoporosis (60). This indicates that obesity management requires a balance between muscle and fat, and we must not only focus on weight loss but also maintain muscle quality. Studies suggest that waist-to-hip ratio (WHR) and free fat mass index (FFMI) may be useful indicators for the early detection of metabolically unhealthy obesity (61). Future obesity management strategies need to move beyond the BMI framework and incorporate additional measurements such as muscle mass, fat distribution, and functional parameters. Research findings indicate that therapeutic interventions and health-related outcomes can be optimized through multi-dimensional and personalized approaches.

### 3.3 Infants and young children

With the advancement of body composition assessment technologies, researchers have begun to study infant dietary intake and changes in body composition, as well as their effects on specific health outcomes such as growth and metabolic development in early life stages (infancy and childhood) (62). There are various techniques for evaluating the body composition of infants. Among them, ADP is widely recognized as the non-invasive gold standard for accurate measurement of fat mass and fat-free mass (62). The reference curves for growth assessment developed according to WHO standards are based on body composition data obtained using ADP, and these curves are useful tools for clinical assessment of growth and nutritional status (63). In addition, DXA or BIA techniques are often used to monitor the body composition of infants. Although DXA and BIA have limitations such as high cost and limited availability, they are still useful methods in resource-constrained settings (64). The choice of technique often depends on the balance between accuracy and feasibility. For research purposes requiring high precision, ADP is preferred. In clinical follow-ups, BIA, despite its limitations in this age group, can provide trends in fluid and soft tissue changes when more advanced methods are unavailable (18). Ultrasound has also been tried to estimate muscle and fat thickness in infants, but its accuracy for total body composition is considered insufficient (27). An infant's body composition reflects their current nutritional status and is also an important predictor of their future health. Studies have shown that the growth rate during infancy and early childhood has a significant impact on body fat accumulation (65). In addition, body composition in infancy is associated with a higher risk of obesity and metabolic disorders in childhood (62, 66). In summary, research and measurement of body composition in infants are growing, with a deeper understanding of the interaction between dietary nutrition, changes in body composition, and health. Future studies need to strengthen the integrated application of various measurement techniques, personalize nutritional interventions, and promote healthy growth of infants while mitigating related risks.

### 3.4 Children and adolescents

Childhood and adolescence are crucial stages for human growth and development. Body composition analysis helps to quantify and accurately measure the nutritional status and disease risk factors of this population, thereby preventing diseases (67). With the development of new measurement technologies, a variety of techniques have been used to estimate the body composition of children and adolescents.

DXA is considered the gold standard for research and clinical samples, but it tends to provide a more accurate estimate of fat percentage in children with higher BMIs and has poor sensitivity in children with low BMIs (8). In addition, different brands of DXA machines and software versions have been shown to produce differences in bone density measurements (68, 69). BIA can be highly inaccurate in estimating fat mass in children classified as normal-weight or underweight using BMI percentiles (70). Given these limitations, the selection of an appropriate method is critical. DXA remains the benchmark for validating other methods and for detailed body composition research in pediatric populations (67). For epidemiological studies or school-basedscreenings, BIA offers a practical solution, but its results must beinterpreted with caution and preferably with population-specific reference data (17). According to reference (71), quantitative ultrasound technology is safe, radiation-free, and portable, making it relatively useful for measuring children's bones. Body composition is closely related to the physiological and pathological states of children and adolescents; for example, exercise habits, sleep quality, and physical activity all affect body composition (72–74). Assessment and cluster analysis of children's body composition can help identify subgroups vulnerable to health risks (75, 76), allowing for timely intervention. In summary, the various techniques available for measuring body composition in children and adolescents each have their own advantages and limitations. In the future, studies should strive to improve age-appropriate standards and measurement procedures, promote the combined application of multiple technologies and the use of AI-assisted analysis, to achieve accurate assessment and effective management of body composition in children and adolescents.

### 3.5 Pregnant women

Body composition research has been widely applied in pregnant women, covering aspects such as assessing physiological changes during pregnancy, predicting pregnancy complications, guiding nutritional interventions, and exploring the association between maternal and infant health (77). Body composition assessment can play an important role in predicting pregnancy complications such as gestational diabetes mellitus (GDM) and macrosomia (78, 79). Body fat measurements and waist-to-hip ratio are helpful for predicting GDM (80). BIA is a commonly used technique in this population due to its safety and ease of use in clinical settings for monitoring changes in body water and fat mass throughout trimesters (80, 81). DXA, while highly accurate, is used more cautiously and typically in research contexts due to radiation exposure concerns, even though the dose is low (77). In the case of twin pregnancies, pre-pregnancy maternal underweight significantly increases the risk of perinatal complications, so nutritional assessment and related management plans are needed (82). The proportion of body fat in pregnant women is affected by dietary fat intake and physical activity; fat accumulation is associated with inactivity and low physical exertion (81). For pregnant women with a normal BMI at conception, increased fat mass is positively correlated with infant birth weight, but the opposite is true for overweight or obese women (83). This shows the relationship between maternal and infant body composition. In conclusion, research on the body composition of pregnant women is essential for maternal and child health management. Body composition assessment of pregnant women improves pregnancy health management through multi-dimensional data analysis. Future research is encouraged to adopt multidisciplinary approaches to gain a deeper understanding of the relationship between pregnant women's body composition and perinatal outcomes, which will help promote precision maternal-fetal medicine.

### 3.6 Menopausal women

Changes in body composition in women after menopause are characterized by increased fat mass (especially in the abdominal area) and decreased muscle mass (84). These changes have a significant impact on both health status and quality of life (85, 86). An increase in body fat percentage is associated with metabolic conditions such as type 2 diabetes and dyslipidemia, physiological conditions such as insulin resistance and inflammation, and an increased risk of cardiovascular diseases (87). Muscle mass loss leads to weakness, reduced grip strength, and impaired mobility, which negatively affects daily activities and overall physical capacity, especially in women who enter menopause at an early age (88). Changes in body composition have also been associated with cognitive decline and mood disorders; in addition, more severe menopausal symptoms such as anxiety and fatigue are significantly correlated with higher body fat percentage and lower muscle mass (89). Physical exercise intervention can help maintain muscle mass while preventing excessive fat accumulation (90, 91). In addition, strong social support from family and friends can help women cope with discomforts such as hot flashes and mood swings, which in turn helps improve body composition and overall quality of life (92). Moreover, studies focusing on the body composition of menopausal women should note the differences in body composition among different races and socioeconomic groups (93). In particular, women with low socioeconomic status, who face high life stress and limited health resources, have more serious body composition abnormalities and higher health risks (94). In conclusion, changes in body composition in menopausal women are a complex multi-factorial phenomenon influenced by physiological hormonal changes, lifestyle, and social and cultural factors. Body composition measurement and intervention strategies can effectively improve the health status and quality of life of menopausal women, providing a scientific basis and management methods for clinical practice.

### 3.7 Older adults

In recent years, interest in evaluating body composition in older adults has increased significantly, with a particular focus on changes in fat mass distribution and muscle quality, which have important implications for health status and disease risk (95). Numerous studies have explored the characteristics of body composition in older adults and their associations with functional status, disease risk, nutritional adequacy, and quality of life. BIA is increasingly accepted as a standard method for measuring body composition and is widely used in clinical and community settings due to its ease of use and non-invasiveness (96). However, many current BIA devices are not designed for diverse populations, including different countries, ethnic groups, and older adults, which may lead to bias in the study population (97). Studies have shown that prediction models optimized for specific elderly groups can improve the accuracy of body composition measurement, which is conducive to community nutritional status monitoring and early identification of sarcopenia (96). Sarcopenia and the gradual loss of muscle mass in older adults are thought to increase the risk of cardiovascular mortality (98). Grip strength is a common measure of muscle strength and physical function; in older adults, increasing lean body mass and maintaining or increasing calf circumference may improve muscle strength, which helps prevent or delay age-related functional decline (99). In addition, the body composition of older adults is largely determined by nutrition, eating habits, lifestyle, and other physical activities (100). The interaction between environmental factors and lifestyle also plays a key role in influencing body composition (101, 102). In summary, research on the body composition of older adults involves improving measurement methods, exploring the relationship between body composition and function, evaluating the impact of environment and lifestyle, and applying these findings in disease management. Future initiatives should go beyond simple outcome measures and adopt large-scale data and multi-omics strategies to establish targeted and flexible body composition assessment and intervention frameworks essential for healthy aging and disease prevention.

### 3.8 Other populations

Research on body composition measurement is not limited to healthy adults or specific disease populations; it involves a variety of population groups, including different ethnic groups, ages, occupations, and individuals with different physiological conditions (103). In addition, body composition assessment is an essential requirement in the management of chronic conditions such as liver disease, cancer, malnutrition, osteoporosis, and metabolic diseases (104). For disease-specific applications, the choice of technique is often driven by the clinical question. For instance, CT is frequently used in oncology to assess muscle wasting (sarcopenia) as a prognostic indicator, as it is often already performed for staging purposes (22, 105). For patients with chronic liver disease, both BIA for bedside assessment and CT/MRI for detailed tissue characterization are valuable for managing nutritional status and predicting outcomes (106–108). Genetic, environmental, lifestyle, and other factors contribute to differences in body composition characteristics among various groups. Therefore, studying the body composition of different groups is clinically and publicly important. Future research should encourage multidisciplinary collaboration, including genetics, environmental science, and social sciences, to develop accurate and feasible body composition measurement methods and evaluation tools suitable for clinical and public health applications.

## 4 Research areas inition measurement

### 4.1 Health management and chronic disease prevention

Body composition evaluation is an important tool for assessing obesity, nutritional status, and health-related risks. It helps accurately measure key indicators such as body fat percentage, fat distribution, and muscle mass (1). Relying solely on BMI is insufficient for a comprehensive evaluation of an individual's metabolic health; factors such as body fat percentage and fat distribution provide more clinical value, including better evaluation of metabolic health and risk stratification (109, 110). Studies have shown that excessive visceral fat accumulation is associated with the development of chronic diseases (58, 59). In this context, DXA and CT are powerful tools for precise quantification of visceral adipose tissue in research and high-risk clinical populations (58). For widespread public health initiatives and personal health tracking, BIA scales and, increasingly, CV/AI-based smartphone apps offer accessible means to monitor trends in body fat and muscle mass, empowering individuals to manage their health proactively (30, 33). Reduced muscle mass is associated with abnormal gait and an increased risk of falls in older adults; timely detection of muscle mass loss through body composition analysis allows for prompt intervention to prevent sarcopenia and falls (111). The muscle-fat ratio also affects cardiovascular health (112). Evaluating body fat and muscle composition is crucial for assessing nutritional status and predicting disease occurrence (13). Maintaining a balanced body water level is important for normal bodily functions; body water composition assessment can reveal abnormalities in kidney function, cardiovascular function, and endocrine system function (113, 114). In addition, it can determine conditions such as edema and dehydration through various quantifiable indicators, which helps in disease management and treatment outcome evaluation (11, 13). In summary, body composition analysis supports the early identification of obesity risks, prevention of metabolic syndrome and chronic diseases, monitoring of muscle mass changes, and maintenance of water balance, thus having great clinical and public health significance. Technological innovations are expected to improve body composition measurement, making personal health management and disease prevention easier.

### 4.2 Clinical medicine

The integration of body composition assessment into clinical medicine has become a common practice for various diseases, including liver disease, tumors, malnutrition, osteoporosis, and metabolic diseases (115). Body composition assessment provides a framework for disease diagnosis, treatment planning, and outcome evaluation (116). The specific technique employed is often tailored to the clinical scenario and available resources. For example, BIA is invaluable for rapid, bedside nutritional assessment in critically ill or cancer patients to estimate fat and protein reserves (12, 117). DXA remains the standard for diagnosing osteoporosis and for detailed body composition profiling in metabolic disorders (6, 115). In surgical oncology, CT scans acquired for routine staging are now opportunistically used to analyze body composition, providing prognostic information without additional patient burden or radiation exposure (22, 105). Accurate body composition assessment is particularly important in nutritional status evaluation, especially in pre-transplant evaluation for liver transplantation; body composition information in children can help develop nutritional strategies and treatment approaches (118). For individuals recovering from injury, surgery, or illness, rehabilitation medicine plays an important role. Monitoring changes in muscle mass can reflect an individual's nutritional status and functional recovery (28, 119), which is necessary for developing effective rehabilitation programs and nutritional guidelines.In this setting, ultrasound is emerging as a practical, bedside tool to track changes in specific muscle groups, complementing functional assessments (28). Changes in body composition parameters (BCPs) have important clinical implications for prognosis. For example, patients with chronic liver disease (CLD) may have sarcopenia and abnormal fat distribution, which affect their quality of life and survival; therefore, changes in muscle and adipose tissue mass have become important prognostic factors in CLD management (106, 107). In oncology, body composition assessment is also important; evidence suggests that preoperative assessment of adipose tissue helps stratify surgical risks (105). Body composition assessment of vulnerable patients, critically ill patients, and cancer patients provides important information on their protein and fat reserves for nutritional support and treatment planning (117, 120), ensuring the development of personalized strategies that significantly improve their nutritional status and clinical outcomes (121, 122). In addition, parameters related to body composition are valuable for monitoring and controlling the side effects of drugs (123). Body composition measurement has enormous benefits in the management of metabolic diseases, rehabilitation medicine, and nutritional support for critically ill and cancer patients. Clinicians can optimize and personalize treatments and rehabilitation strategies by continuously monitoring changes in muscle and fat, thereby improving outcomes and quality of life. In the future, body composition analysis will be more closely integrated with clinical practice, further improving the precision of medicine.

### 4.3 Exercise science and physical training

With the increasing prevalence of chronic diseases, the role of exercise science and physical training in disease prevention and health promotion has become increasingly clear (124). A number of studies have shown that appropriate levels of physical activity and exercise programs can significantly improve body composition, as well as physical ability and overall quality of life, providing a good theoretical and practical basis for the development of exercise science and physical education (125). Assessing the impact of exercise interventions on body composition is crucial for developing science-based exercise recommendations. For example, combining high-intensity interval training with aerobic exercise may promote fat oxidation and reduce body fat percentage in individuals with obesity and metabolic syndrome (126). Exercise training can also improve physical fitness and body composition in patients with chronic diseases; different types and sequences of physical exercise have different effects on specific aspects of body composition (127–129). Individuals with diseases such as cirrhosis or cancer can improve muscle quality and body composition through exercise programs and dietary adjustments, leading to better physiological function, including improved strength, endurance, and metabolic efficiency (130, 131). In rehabilitation settings, muscle mass gain is closely related to functional rehabilitation in elderly fracture patients, stroke survivors, and post-stroke recovery (132–134). Exercise interventions have different effects on body composition in different age and gender groups; for example, perimenopausal women and overweight adolescents show unique body composition changes in response to exercise training (135, 136). Future research must further clarify how different types, intensities, frequencies, and volumes of exercise affect body composition and function in different populations, with the goal of improving personalized exercise prescriptions and the scientific application of exercise training in rehabilitation and health care.

## 5 Future research directions

Currently, medical devices such as CT, DXA, and BIA are the gold standards for body composition measurement. While these methods are highly accurate and reliable, they are limited by high costs, complex operating conditions, and the need for personnel training, making them unsuitable for public health management and routine monitoring. On the other hand, smartphone applications based on CV and AI are cost-effective, user-friendly, and capable of continuous monitoring, making them indispensable in health, fitness, and chronic disease management. Future research will likely focus on the following areas.

### 5.1 Dynamic monitoring and trend insights

Traditional static assessments focus on measuring parameters such as body fat percentage and muscle mass at a single time point but do not consider changes in body composition over time (137). In recent years, dynamic monitoring has received increasing attention, as body composition is becoming more important in health management, especially for chronic disease management, exercise rehabilitation, and elderly health maintenance. A recent study on gait in older adults found a correlation between several dynamic gait indicators and static body metrics: dynamic indicators were positively correlated with muscle mass and negatively correlated with body fat, indicating that muscle mass plays a key role in exercise adaptation (138). With the widespread use of mobile health applications, user needs have shifted from requiring a single metric (e.g., step count or heart rate) to multiple metrics (139), indicating a growing demand for comprehensive insights rather than discrete data points. Research is expected to advance from one-time body composition measurements to long-term dynamic measurements, focusing on trend analysis of multiple indicators. Applications and smart devices will be key tools to achieve this goal. By adopting such approaches, we can not only assess health status more comprehensively but also prevent chronic diseases, providing a scientific basis and technical support for sports rehabilitation and elderly health.

### 5.2 Multimodal fusion becomes standard

With the development of various body composition measurement technologies, existing methods based on a single data collection approach can no longer meet the accuracy and comprehensiveness requirements of clinical and research applications (140). Therefore, multimodal fusion technology, which integrates information from multiple sources to improve the accuracy of body composition assessment and enable comprehensive analysis of features from different datasets, has become an essential method (141). For example, mobile devices equipped with optical radar and ToF sensors can capture high-precision 3D body contours, making volume and fat measurements more accurate. The increasing use of heart rate and heart rate variability data from wearables allows for the tracking of an individual's metabolic status and autonomic regulation, which is valuable for professionals assessing muscle mass, fat mass, and overall metabolic health. Recent studies on the relationship between heart rate variability and body composition in obesity, sports rehabilitation, and metabolic disorders have shown good clinical application prospects (142). In conclusion, the integration of multiple modalities in body composition measurement is an inevitable trend. In the future, real-time monitoring of body composition will become a reality: advanced 3D data from mobile LiDAR systems and sensors will be combined with biometric data from wearables, overcoming the limitations of individual data types. These developments will make body composition measurement more comprehensive, dynamic, and precise, establishing a standard technological path for future intelligent healthcare and health management.

### 5.3 Concentration on specific application areas

As body composition measurement technology advances, it will shift from being “comprehensive and extensive” to more context-specific. In fitness and bodybuilding, accurate monitoring of muscle mass changes and subcutaneous fat distribution is crucial (143). In nutrition, studying the effects of supplements on body composition and metabolism will be an important research area (144, 145). For elderly health, evaluating sarcopenia is a key topic in body composition research, as it significantly affects the physical capacity and quality of life of older adults (146). Continuous monitoring of body composition in pregnant women is essential to promptly identify nutritional deficiencies that may affect the mother and fetus, enabling early intervention and preventing complications (147). Body composition assessment for liver transplant patients helps evaluate muscle wasting and nutritional status (108). Furthermore, the application of advanced body composition monitoring technologies provides new ways to improve disease prognosis and guide treatment adjustments in the management of various chronic conditions, including those related to the neuromuscular and immune systems (148). In summary, body composition measurement technology has moved from a holistic approach to focused development in specific areas such as fitness, geriatric health, maternal and child health, and clinical support. In the near future, body composition measurement will play a more important role in personalized health management and precision medicine, providing more accurate and convenient solutions for specific professional fields.

## 6 Conclusion

Body composition assessment technology is currently at a crossroads of rapid technological integration and innovation. In general, new technologies will prioritize the joint application of multiple methods, intelligent data processing, and standardization across fields to achieve high precision, efficiency, and versatility in measurement. Advancing this progress will help us better understand human physiology and improve the standards of precision medicine and health management. Ultimately, this will not only benefit a wide range of patients and healthy individuals but also promote the further development of medical science and public health.
